# Ultra-Performance Liquid Chromatography Coupled with Mass Metabolic Profiling of *Ammi majus* Roots as Waste Product with Isolation and Assessment of Oral Mucosal Toxicity of Its Psoralen Component Xanthotoxin

**DOI:** 10.3390/metabo13101044

**Published:** 2023-09-29

**Authors:** Noha Fathallah, Mona El Deeb, Amany A. Rabea, Alshaimaa M. Almehmady, Hanaa Alkharobi, Sameh S. Elhady, Noha Khalil

**Affiliations:** 1 Department of Pharmacognosy and Medicinal Plants, Faculty of Pharmacy, Future University in Egypt, Cairo 11835, Egypt; 2 Department of Oral Biology, Faculty of Oral and Dental Medicine, Future University in Egypt, Cairo 11835, Egypt; mona.eldeeb@fue.edu.eg (M.E.D.); amany.ahmed@fue.edu.eg (A.A.R.); 3 Department of Pharmaceutics, Faculty of Pharmacy, King Abdulaziz University, Jeddah 21589, Saudi Arabia; amnalmehmady@kau.edu.sa; 4 Department of Oral Biology, Faculty of Dentistry, King Abdulaziz University, Jeddah 21589, Saudi Arabia; halkharobi@kau.edu.sa; 5 Department of Natural Products, Faculty of Pharmacy, King Abdulaziz University, Jeddah 21589, Saudi Arabia

**Keywords:** *Ammi majus* roots, Apiaceae, metabolites, ADME, boiled egg, histopathological studies, Xanthotoxin, oral safety, health care

## Abstract

*Ammi majus*, a well-established member of the Umbelliferae (Apiaceae) family, is endogenous to Egypt. The main parts of this plant that are used are the fruits, which contain coumarins and flavonoids as major active constituents. The roots are usually considered by-products that are discarded and not fed to cattle because of coumarins’ potential toxicity. The goal of this study was to ensure the sustainability of the plant, investigate the active metabolites present in the roots using UPLC/MS-MS, isolate and elucidate the major coumarin Xanthotoxin, and predict its oral bioavailability and its potential biological impact on tongue papillae. The results revealed coumarins as the dominant chemical class in a positive acquisition mode, with bergaptol-*O*-hexoside 5%, Xanthotoxin 5.5%, and isoarnoittinin 6% being the major compounds. However, phenolics ruled in the negative mode, with *p*-coumaroyl tartaric acid 7%, 3,7-dimethyl quercetin 6%, and hesperidin 5% being the most prominent metabolites. Fractionation and purification of the chloroform fraction yielded Xanthotoxin as one of the main compounds, which appeared as white needle crystals (20 mg). ADME studies for oral bioavailability were performed to predict the potential properties of the compound if used orally. It was noted that it followed Lipinski’s rule of five, had just one parameter outside of the pink area in the radar plot, and was detected inside the threshold area using the boiled egg approach. In vivo, histopathological studies performed on rats showed a notable decrease in the tongue’s keratin thickness from an average of 51.1 µm to 9.1 µm and an average of 51.8 µm to 9.8 µm in fungiform and filiform cells, respectively. The results indicated that although Xanthotoxin is a well-known medical agent with several potential therapeutic activities in oral therapy, it may cause a destructive effect on the structure of the specialized mucosa of the tongue.

## 1. Introduction

Herbs are gaining more attention day by day as they offer natural defense against certain illnesses. The World of Health Organization promotes medical herbs and plants to replace or minimize chemical use as part of the global trend to get back to nature [1]. Numerous investigations on non-traditional raw ingredients were conducted, including an array of plant and animal by-products. For centuries, the world’s agriculture waste management system was described by the 3 I’s (inefficient, irregular, and inadequate), leading to a critical increase in environmental pollution [2]. The improper handling and use of these agricultural wastes may eventually result in the loss of a potentially significant economic resource of bioenergy and valuable products [3]. Thus, it was deemed necessary to explore new and alternative methods for utilizing these potentially valuable resources and changing people’s behavior toward them [4]. In Egypt today, 18% of agricultural wastes are used directly as fertilizer [3], with 30% more used as animal food, and the remainder is directly burnt on the fields or applied as a heating source in small villages [4]. *Ammi majus* (Apiaceae) is commonly grown in the tropics, subtropics, and temperate regions, as well as in Egypt, and can also be found in wild forms. [5]. It is a biennial plant that grows annually [5,6]. *A. majus* was used in folk medicine long before it was accepted medically. It was originally used in skin disorders like psoriasis and vitiligo, as a diuretic for kidney stones and infections of the urinary tract system, as an emmenagogue to control menstruation, and in other conditions [7,8]. The fruits of *A. majus* are now considered the main organ used in modern medicine due to their active metabolites, furanocoumarins like Xanthotoxin, imperatorin, marmesin, and bergapten [9,10,11]. However, the roots of the plant are considered a by-product that cannot be fed to cattle due to the potential toxicity of the furanocoumarin content [11]. According to the Egyptian Herbal Monograph [12], and our knowledge, this is the first attempt to reveal the chemical profile of the active metabolites found in the roots.

Xanthotoxin, also known as 8-methoxypsoralene, is a linear furanocoumarin [13] that is present mainly among the members of the family Umbelliferae (Apiaceae). It was previously isolated from *Petroselinum hortense*, *Heracleum persicum*, *Pastinaca sativa*, and a variety of other plants [9,14,15]. It is considered the most important coumarin isolated from *A. majus* and is currently used topically to treat skin illnesses such as vitiligo and psoriasis [15,16,17]. In the past few years, scientific research has reported the significant potential pharmacological activities of Xanthotoxin [9,17,18,19,20,21,22,23,24], which may need its administration through oral or intravenous (IV) routes rather than topical administration. However, only a few studies have investigated the safety margin of oral consumption of the compound [22,25,26]. Xanthotoxin was previously reported to have a toxic effect on the histology of several tissues, including the liver, testes, and adrenal glands [27,28].

This study aimed to investigate the active metabolites present in the roots of *A. majus* by ultra-performance liquid chromatography coupled with mass (UPLC-MS/MS), which serves as an important source of pharmacologically active metabolites, as well as the isolation of one of its major furanocoumarins, Xanthotoxin, and assess its safety profile on the tongue papillae of *albino* rats as an example of oral mucosal tissue. To our knowledge, this is the first attempt to evaluate the safety margin of Xanthotoxin on the tongue papillae.

## 2. Materials and Methods

### 2.1. Plant Materials

Roots of *Ammi majus* L. cultivated in an open field area (15 × 75 m) were collected from farmlands of the Medicinal, Aromatic, and Poisonous Plants Experimental Station of the Faculty of Pharmacy, Cairo University, Egypt, in June 2020. Dr. Mokhtar Bishr, Technical Director of the Arab Company for Pharmaceuticals and Medicinal Plants (Mepaco Co.), Egypt, identified plant samples, and voucher specimens were deposited at the Pharmacognosy research lab at the Faculty of Pharmacy, Future University in Egypt, with the number RD-235-018. The roots were separated, powdered, and stored in an amber glass container for further use.

### 2.2. Chemicals and Solvents

All the chemicals/solvents were bought from Pio-chem firm in Cairo, Egypt, and were of excellent purity. HPLC-grade methanol was obtained from Merck (Merck KGaA, Darmstadt, Germany).

### 2.3. Extraction of the Plant Material

The total *A. majus* ethanolic extract (TAE) (75 g) was obtained by extracting the air-dried roots of *A. majus* (500 g) three times, consecutively, with ethanol 95% (3 × 500 mL) at room temperature until complete exhaustion of the roots [7,29,30,31]. The extract was then evaporated under vacuum using a rotary evaporator (Buchi^®^ Rotavap R-114, Flawil, Switzerland) at a temperature not exceeding 50 °C and stored in the refrigerator till usage.

### 2.4. UPLC-MS/MS Identification

The ethanolic extract sample (100 µg/mL) was prepared in MeOH (HPLC-grade) and filtered through a membrane disc filter (0.2 µm) before analysis. A total of 20 µg/mL of TAE was then investigated utilizing the positive and negative ion acquisition modes of the ESI-MS. They were analyzed on an XEVO TQD triple quadruple instrument (Waters Corporation, Milford, MA, USA) mass spectrometer. The column specifications were as follows: ACQUITY UPLC-BEH C18 1.7 µm −2.1 × 50 mm. The flow rate used was 0.2 mL/min. The solvent system consisted of (A) water containing 0.1 % formic acid and (B) acetonitrile containing 0.1 % formic acid using a gradient elution system starting from the gradient mobile phase, starting from (90% A: 10% B), till (0% A: 100% B). The mass spectrometer conditions were performed according to [32,33] as follows: source temperature: 150 °C, cone voltage: 30 eV, capillary voltage: 3 kV, temperature: 440 °C, cone gas flow: 50 L/h, and de-solvation gas flow: 900 L/h. Mass spectra were identified in the ESI between the *m*/*z* 100 and 1000 according to [34], using the Maslynx 4.1 software, and the peaks and spectra were processed and tentatively identified by comparing their retention times (Rt) and mass spectra to the reported data.

### 2.5. Fractionation and Purification

Fractionation was performed on the total TAE (75 g) using *n*-hexane (4 × 500 mL), followed by chloroform (4 × 500 mL) and ethyl acetate EtOAc (4 × 500 mL). The three fractions were individually evaporated under vacuum till dryness was achieved to give hexane fraction dry weight (10 g), CHCl_3_ (5.57 g), EtOAc fraction (3.54 g), and ethanol (8.21 g). The CHCl_3_ was chosen for further isolation based on Thin-Layer Chromatography (TLC) spotting results and by comparing the published data [9,35,36]. Further fractionation was performed under Vacuum Liquid Chromatography (VLC). Each of the eluates (40 mL) were further investigated using TLC, silica gel 60F 254, by different solvent systems with various polarities, and then purified using the Puriflash 4100 system (Interchim; Montluçon, France), consisting of 25 g flash-NP column (30 µm), a mixing HPLC quaternary pump, a PDA–UV–Vis detector 190–840 nm, a fraction collector, and a sample loading module. For system control and process monitoring, Interchim Software 5.0 was used. The purified samples were collected, and then the solvents were removed by evaporation. TLC silica gel 60F 254 was used for the comparison of the R_f_ values to identify the compounds.

### 2.6. Studies on Pharmacokinetics and ADME (Absorption, Distribution, Metabolism, and Excretion)

To ascertain whether Xanthotoxin had the potential to be a promising pharmaceutical drug, the Absorption, Distribution, Metabolism, and Excretion (ADME) and Pharmacokinetic Studies were conducted using SWISSadme [37] (Swiss Institute of Bioinformatics online source), link: www.swissadme.ch, accessed on 4 September 2023. Xanthotoxin physicochemical characteristics for oral bioavailability were identified by the Swiss ADME molecules’ bioavailability radar. The pink region represents the ideal spaces for six physicochemical properties, such as polarity, size, solubility, lipophilicity, flexibility, and saturation, for the oral bioavailability of the representative compound. Swiss ADME was also utilized in anticipating the blood barrier and GIT absorption of the compound.

### 2.7. Biological Study Design

The animal handling adhered to the guide for the care and use of laboratory animals (NIH publication No. 85-23, revised 1996) [38]. The protocol was revised and approved by the research ethics committee of the faculty of Pharmacy, Future University in Egypt on 14 November 2022, with the number (REC-FPFUE-17/135). Four male albino rats weighing 200–250 g were housed in separate cages at the animal house of Future University in Egypt. They were kept under controlled temperature, humidity, and dark/light cycles and had free access to food and water throughout the experimental period. This was performed under the supervision of a specialized veterinarian. The rats were divided randomly into two equal groups: In the control group, rats were orally administered with 2 mL of saline once/day using an intragastric gavage needle for 5 successive days. In the experimental group, rats received a daily single dose of 200 mg/kg of Xanthotoxin dissolved in saline, by intragastric gavage needle for 5 successive days [39]. By the end of the experiment, the rats were sacrificed using sodium thiopentane, 200 mg/kg for each rat, after cervical dislocation [40], and the tongues were dissected out and processed for routine histological examination using hematoxylin and eosin (H&E) staining [41]. The sections were examined by an inverted metallurgical microscope (inverted LM, Olympus^®^, BX40F4, Tokyo, Japan) and photographed at 200× magnification.

### 2.8. Statistical Analysis

Recorded data were analyzed using the statistical package for social sciences, version 23.0 (SPSS Inc., Chicago, IL, USA). The quantitative data were presented as mean ± standard deviation and ranges. Data were explored for normality using Kolmogorov–Smirnov and Shapiro–Wilk tests. The following tests were performed: Independent samples *t*-test of significance was used when comparing between two means. The confidence interval was set to 95%, and the accepted margin of error was set to 5%. So, the *p*-value was considered significant as follows: *p*-value ≤ 0.05 was considered significant, *p*-value ≤ 0.001 was considered highly significant, and *p*-value > 0.05 was considered insignificant.

## 3. Results

### 3.1. Ultra-Performance Liquid Chromatography Coupled with Mass (UPLC-MS/MS) Profiling

**Figure 1:**
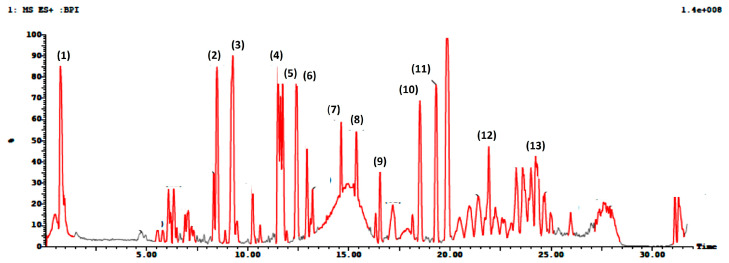
Total ion UPLC-ESI/MS chromatogram in positive acquisition mode of *A. majus* roots.

**Figure 2:**
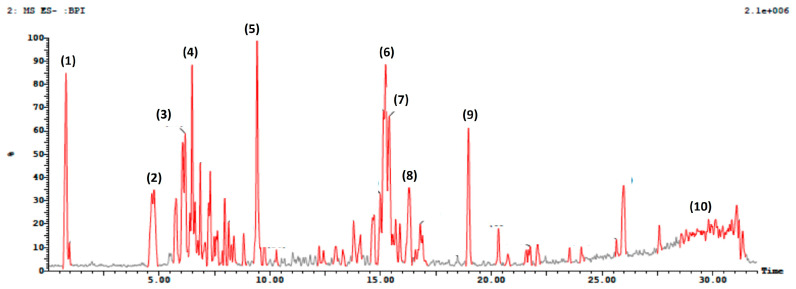
Total ion UPLC-ESI/MS chromatogram in negative acquisition mode of *Ammi majus* roots.

**Figure 3:**
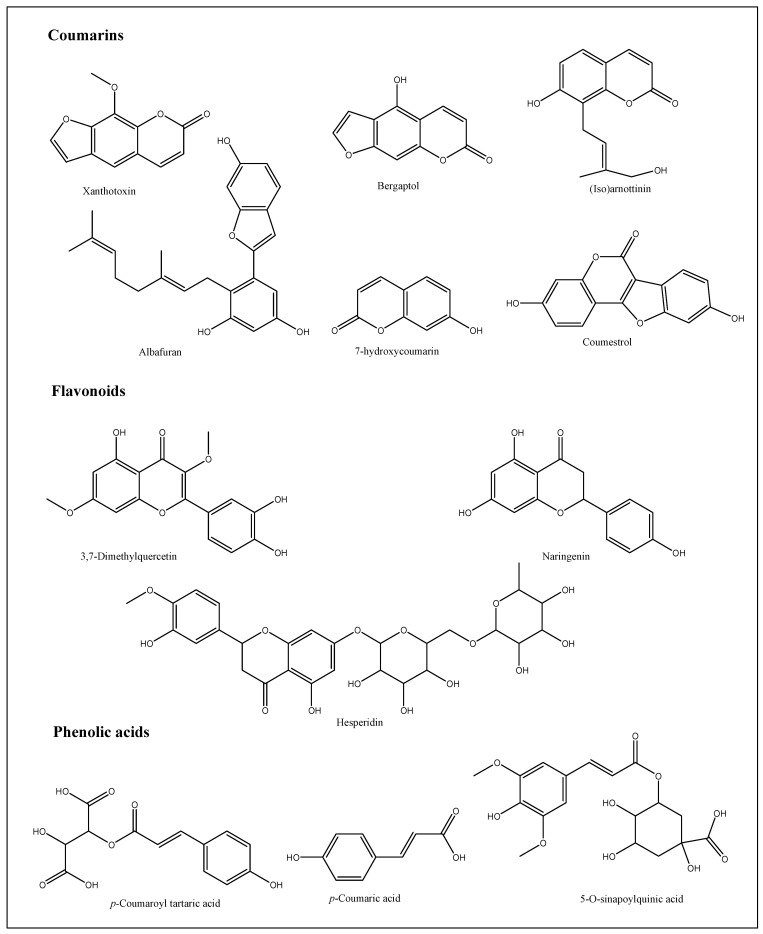
Two-dimensional structures of the main detected compounds of *Ammi majus* L. by UPLC/MS analysis.

UPLC-ESI-MS/MS analysis gave a comprehensive metabolite profile of the *A. majus* roots. Due to the different ionization needs of the individual chemicals, the extract was studied in both negative and positive ion ionization modes. A typical UPLC-ESI-MS chromatogram of the extract in positive and negative ion ionization modes is shown in Figure 1 and Figure 2, along with the identification of 26 chemicals in the root extracts (Table 1 and Table 2). The findings showed that coumarins predominated in the positive ionization mode, while flavonoids and phenolic compounds, such as flavonol sulfates, flavonol glycosides, and isoflavone aglycones and glycosides, predominated in the negative ionization mode with a distinctly greater percentage. A scheme showing the identified compounds is illustrated in Figure 3. After observing the fragmentation patterns of some compounds, it was noted that the typical coumarin fragmentation was spotted [42] with the loss of CO_2_ followed by the opening of the ring and the loss of C_2_H_2_. The major compounds identified in the positive ion were Xanthotoxin and bergaptol-O-hexoside. Meanwhile, the fragmentation pattern of some compounds resembled that of flavonoids and flavonoids glycosides, the majority of which revealed a typical fragmentation pattern with the loss of CO and CH_2_O groups followed by a loss of glycosidic linkage and a loss of H_2_O, as illustrated by Cuyckens and Claeys [43]. Fragments resembling this pattern were detected in both acquisition modes, with the relative abundance being higher in the negative mode. Among the flavonoids identified were dihydroxyflavone, naringenin, and hesperidin. It was noted that most of the active metabolites resemble those found in the fruits and aerial parts, which is advantageous regarding the amount required to be used in medicine every year, thus ensuring the sustainability of the plant.

### 3.2. Structural Isolation and Identification of the Compound

After fractionation and purification, three major fractions were obtained (*n*-hexane, CHCl_3_, and EtOAc). The chloroform fraction was chosen for further isolation after observing the coumarin spots on the TLC under UV [54]. They gave characteristic fluorescence with UV and significant colors after the treatment with ammonia vapor. Five chemicals were obtained from the chloroform fraction upon purification. Our aimed compound, Xanthotoxin (20 mg), appeared as white needle crystals, and its identification was confirmed by comparing the R_f_ value with a standard isolated and identified by [9]. It was chosen for evaluating its safety profile as it is one of the major compounds found in the roots plus it has more medical importance when compared to all other *A. majus* active metabolites.

### 3.3. The Lipinski’s Rule of Five, ADME, and Boiled Egg Techniques

**Figure 4:**
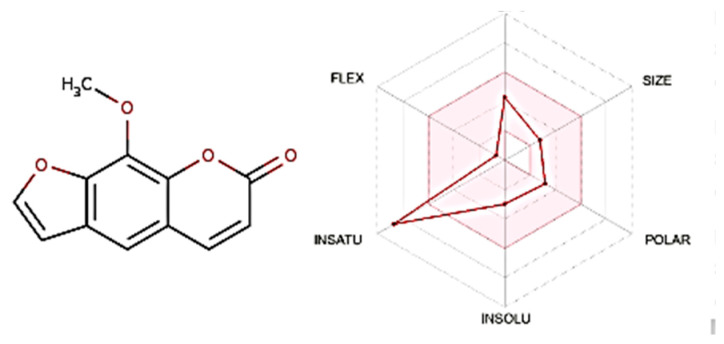
Radar plot of Xanthotoxin: POLAR (polarity), LIPO (lipophilicity), INSOLU (solubility), FLEX (flexibility), and IN-SATU (saturation). Lipophilicity: XLOGP3 between −0.7 and +5.0; solubility: log S < 6; sizes: MW between 150 and 500 g/mol; saturation: fraction of carbons in the sp3 hybridization > 0.25; and polarity: TPSA between 20 and 130 Å; flexibility: < 9 rotatable bonds.

**Figure 5:**
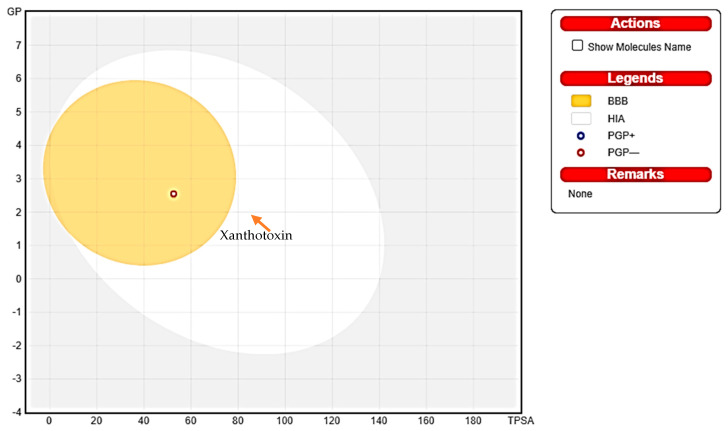
Xanthotoxin evaluation using boiled egg method for BBB and GIT absorption. WLOGP is 2.55 and the TPSA is 53.58 Å. *N.B: Boiled egg 2D graphical representation; the yolk area represents the molecules that can passively permeate through the blood-brain barrier (BBB), whereas the molecules located in the white region are predicted to be passively absorbed by the gastrointestinal (GI) tract*.

Physiochemical parameters of Xanthotoxin were evaluated by applying ADME and Lipinski’s rule of five, which help make decisions on the approval of potential compounds for biological systems [69,70]. As observed in Table 3, it was observed that Xanthotoxin passed the first step to become an oral medicinal drug by complying with Lipinski’s parameters; however, as seen in Figure 4, regarding the radar plot bioavailability technique, it is predicted to not be fully orally bioavailable, as it has one parameter outside the pink (bioavailability) area. Another method to evaluate absorption and penetration of the drugs is the boiled egg technique, where Xanthotoxin is predicted to be a brain-penetrant found in the yolk as seen in Figure 5, with WLOGP 2.55 and TPSA 53.58 Å, and it is a non-substrate of P-glycoproteins (PGP) (red dot), yet it remains within the acceptable range as all the readings were found in the threshold area (WLOGP ≤ 5.88 and topological surface area TPSA ≤ 131.6) [70]. Because of its attractive ADME characteristics, Xanthotoxin was explored further to evaluate its safety for human oral consumption.

### 3.4. Histological Results

**Figure 6:**
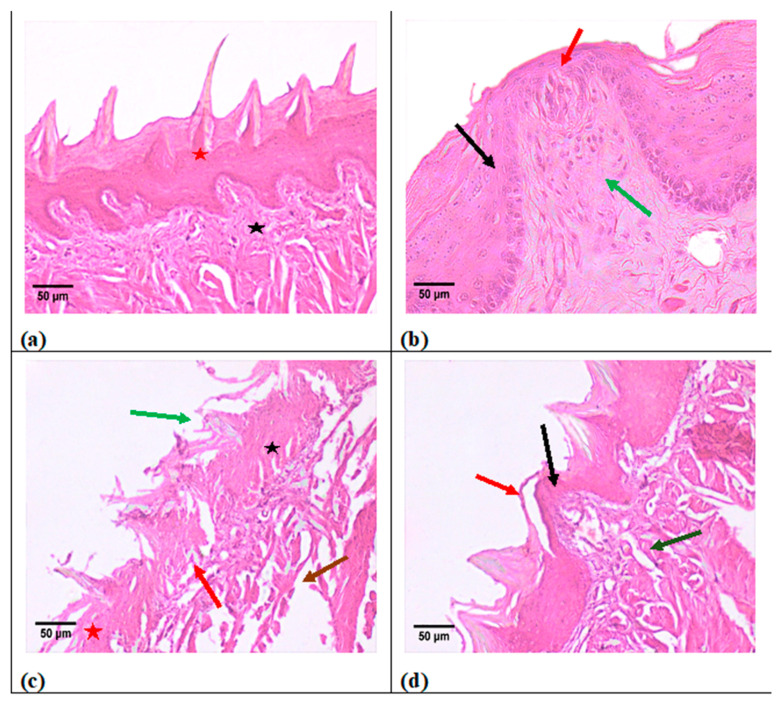
Histological photomicrographs of Group 1 control group show the following: (**a**) well-arranged and defined epithelial cells with keratin tips (red asterisks), and arranged connective tissue that is well formed (* in black); (**b**) mushroom-shaped fungiform papilla with well-defined keratinized stratified squamous epithelium layers (black arrow), single well-defined barrel-shaped taste bud (red arrow), and well-defined connective tissue papillae (green arrow); (**c**) thinning of stratified squamous epithelium (red asterisk), loss of keratin tips over some papillae (green arrow), indefinite epithelial cell boundaries (black asterisk), irregular epithelial rete pegs (red arrow), and degenerated underlying connective tissue (brown arrow); (**d**) destructed fungiform papilla with degenerated intraepithelial taste bud (black arrow), detached keratin layer (red arrow), degenerated lamina propria with massive vacuolization (green arrow), and hematoxylin and eosin (H&E) staining, H&E, Orig. Mag. ×200.

**Figure 7:**
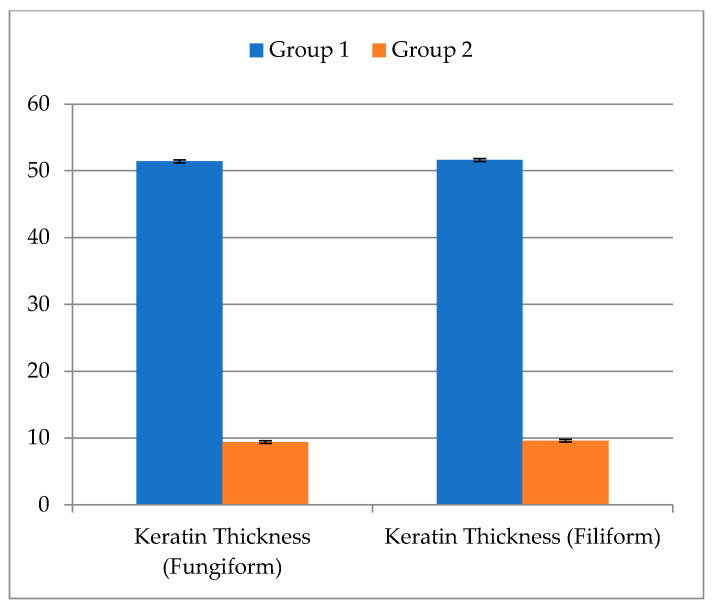
Comparison between Group I and Group 2 according to keratin thickness in fungiform cells and: filiform cells.

The control group histological analysis revealed significant and consistently aligned flame-shaped filiform papillae with distinct cell layers of keratinized stratified squamous epithelium covering (51.1 µm). It was found that the underlying connective tissue was well organized and structured (Figure 6a). The fungiform papilla displayed a recognizable mushroom shape, rising above the tongue’s surface and covered in keratinized stratified squamous epithelium (51.8 µm). In the most external epithelial layer, a thin, homogeneous layer of keratin was seen. On the dorsal surface, one distinct taste bud in the form of a barrel was seen. There was a well-identifiable connective tissue papilla that made up the lamina propria (Figure 6b). However, in the experimental group, the filiform papillae in this group had a disrupted pattern upon histological analysis, and some of the papillae had lost their keratin tips. Keratinized stratified squamous epithelium showed degeneration with an evident thinning decline to 9.1 µm and ambiguous epithelial cell borders. Furthermore, irregular epithelial rete pegs were seen. Degeneration of the underlying connective tissue was significant (Figure 6c,d). It was found that the intraepithelial taste bud had deteriorated and the fungiform papilla had been destroyed to reach 9.1 µm. Massive vacuolization caused the lamina propria’s connective tissue to deteriorate. As illustrated in Figure 7, the decline in thickness of keratin was more than 5-fold, indicating massive destruction of keratin thickness.

### 3.5. Statistical Analysis

As seen in Table 4 and Figure 7, regarding fungiform and filiform cells, Group 2 revealed a significant mean value decline in keratin thickness (9.40 ± 0.22 µm and 9.60 ± 0.22 µm), compared to Group 1 (51.40 ± 0.22 µm and 51.60 ± 0.22 µm, respectively, with a *p*-value (*p* < 0.001).

## 4. Discussion

Agricultural waste is defined as the by-products of the production and processing of agricultural products that may include components that are useful to humans [71].

*Ammi majus* species is one of the most promising sources of furocoumarins. More than 20 furocoumarins (psoralens) were found in the fruits with at least eight medically important coumarins and coumarin glucosides, such as Xanthotoxin, imperatorin, marmesin, ammirin, and isoarnottinin, etc. [9]; however, other parts of the plant, such as the aerial parts, roots, and flowers, are discarded as by-products in large quantities, causing environmental pollution.

The lack of reports on the roots of *A. majus* provoked this investigation to evaluate them as a potential source of secondary metabolites and thus aid in the sustainability of this plant and in decreasing the pollution of the environment [72].

In this study, rapid and efficient ultra-high-performance liquid chromatography coupled with mass spectrometry (UPLC/MS-MS) was chosen to give a complete profile of the compounds present in the roots as it is sensitive and accurate, plus it offers separation in a shorter development and analysis time than the conventional LC/UV [73]. The chromatograms demonstrated numerous but different compounds in both positive and negative acquisition modes. Coumarins like Xanthotoxin and (iso)arnottinin were the most abundant (5.5% and 6%) in the positive mode. Many other coumarins were identified as bergaptol-*O*-hexoside, dihydrochalcone (phloretin), coumestrol, and bergaptol. However, the negative acquisition mode revealed mainly flavonoids and phenolics with *p*-coumaroyl tartaric acid and 3,7-dimethylquercetin being the most abundant (7 and 6%), respectively. Coumarins are known to possess [74] a remarkable array of biochemical and pharmacological actions, suggesting that they may significantly affect the function of various mammalian cellular systems.

Xanthotoxin is a well-established coumarin with recorded medical value as an anti-inflammatory, anticonvulsant, anticancer, etc. [9,20,75,76]. It is most currently used topically to treat skin conditions like psoriasis and vitiligo [45,77]. It was used topically and orally, alone or with other coumarins, in a type of treatment called photochemotherapy [78]. It has also been studied as a potential anticancer agent against HepG-2 cancer [9]; thus, it was deemed necessary to isolate the compound, predict the oral bioavailability, and study and inspect the oral safety profile of this particular compound.

To isolate Xanthotoxin, fractionation and purification of the root extract were performed as mentioned in the Materials and Methods section. It appeared as white needle crystals and was confirmed by comparing it to the standard isolated by Issa et al. [9] using TLC.

Before starting the in vivo studies, the ADME approach was applied to Xanthotoxin using Swiss ADME for the prediction and modeling of its potential oral bioavailability.

It is well known that experimentally observed or computationally predicted ADME data offer crucial insights into how a drug will ultimately be metabolized or absorbed by the body [79]. Three methods were applied to the compound, namely Lipinski’s rule of five, radar plot, and boiled egg. Lipinski’s rule of five and radar plot are common practices to evaluate a chemical compound’s drug-likeness and determine whether it has the qualities to be an orally active medicine in humans [80]. The boiled egg, also known as the brain or intestinal estimated permeation technique, was used because it just uses WLOGP and TPSA as physicochemical descriptors for lipophilicity and apparent polarity, respectively. This model is theoretically simple, and it was precisely created with reliability and statistical significance in mind [70].

Xanthotoxin revealed a promising oral bioavailability profile by following the parameters of Lipinski’s rule of 5. In this, five out of six radar plot parameters were located in the pink area (bioavailability), and the penetration of the BBB was within the threshold range, indicating a potential benefit of formulating the drug as an oral dosage form.

Further, in vivo studies were performed on Xanthotoxin, as it was tested on the tongue papillae of rats. The thickness of keratin in two types of lingual cells, namely fungiform and filiform, was evaluated, both with and without the application of the compound. Those cells are known to act as proxy structures used for oral stimuli detection and transmission [81].

As seen in Figure 6, it was noticed that after exposure to the compound, a disturbed pattern of the filiform and fungiform papillae, loss of some keratin tips, and degeneration with obvious thinning and indefinite epithelial cell boundaries of keratinized stratified squamous epithelium were observed. This foregoing observation matches that obtained by Farhadi et al., who recorded disorganized ovarian follicles, cell injury, mild hepatic degenerative changes, and deterioration of the seminiferous tubules in mice testes after exposure to Xanthotoxin (methoxsalen) [27,28]. A possible explanation for the damaging effect of Xanthotoxin may be related to its byproducts, which display toxic effects on the cell membrane and nucleic acids. It was confirmed that methoxsalen could interact with cellular DNA, causing chromosomal aberrations and gene mutations. It also promotes cell injury and apoptosis in various tissue types [27,28]. Therefore, the potential risk of the oral intake of Xanthotoxin must be assessed to ensure its safe use in medicinal therapy.

## 5. Conclusions

*Ammi majus* root chemical profiling showed a variety of biologically active metabolites that can be considered potential drug sources. Not only can the root usage ensure the sustainability of the plant, but it can also decrease the pollution of the environment and ensure a continuous supply of the coumarins used in medicine. Xanthotoxin is a well-established coumarin known for its medical value, especially in vitiligo and psoriasis. In our study, this compound was isolated, identified, and evaluated for its pharmacokinetics and oral safety. It was shown that it may cause a destructive effect on the structure of the specialized mucosa of the tongue by affecting the keratin thickness in filiform and fungiform cells. It is important to note that there is still limited available clinical data from clinical trials of Xanthotoxin, which restricts its use in medicine. In the future, more thorough research should be carried out to ensure its safe use in any oral therapy and whether the oral cavity damage is reversible or not.

## Figures and Tables

**Table 1:** Peak assignments of the ethanolic extracts of *Ammi majus*, L roots via UPLC-ESI-MS/MS in positive ionization mode.

Peak ID	Name	Rt (min)	Molecular Formula	Area %	[M+H]+	Fragmentation (MS2)	References
1	Bergaptol-*O*-hexoside	0.7	C_17_H_16_O_9_	5%	365	364, 202, 184, 104	[44]
2	Xanthotoxin	8.4	C_12_H_8_O_4_	5.5%	217	216, 202, 189, 185, 174, 161	[45]
3	(Iso)arnottinin	9.2	C_14_H_14_O_4_	6%	247	246, 228, 213, 175, 174, 145, 131, 70	[46]
4	Di hydro chalcone (Phloretin)	11.5	C_15_H_14_O_5_	4%	275	274, 269,209	[47]
5	Bergaptol	12.4	C_11_H_6_O_4_	4%	203	203, 175, 157, 147, 131, 129, 103, 91	[48,49]
6	*p*-Coumaroyl glycolic acid	12.9	C_11_H_10_O_5_	2%	223	208, 165	[50]
7	Taxifolin	14.6	C_15_H_12_O_7_	4.5%	305	305, 123, 95	[51]
8	Pelargonidin	15.3	C_26_H_29_O_14_	4%	566	566, 334	[52]
9	Gallic acid 4-O-glucoside	16.5	C_13_H_16_O_10_	1%	333	333, 332,122	[53]
10	Coumestrol	18.5	C_15_H_8_O_5_	3.5%	269	269, 268, 221	[54,55]
11	*p*-Coumaric acid (*p*-Coumaroyl tartaric acid)	19.3	C_13_H_12_O_8_	3.5%	297	297, 256,	[56]
12	*p*-Coumaroyl malic acid	21.9	C_13_H_12_O_7_	2%	281	281, 247	[56]
13	3,5-Diferuloylquinicc acid	23.2	C_27_H_28_O_12_	2%	544	383, 355, 337, 323	[57,58]

**Table 2:** Peak assignments of the ethanolic extracts of *A. majus*, L roots via UPLC-ESI-MS/MS in negative ionization mode.

Peak ID	Name	Rt (min)	Molecular Formula	Area %	[M-H]^−^	Fragmentation (MS2)	References
1	Albafuran	0.8	C_24_H_26_O_4_	4%	377	377, 361, 345, 329	[59]
2	7-Hydroxycoumarin	4.7	C_9_H_6_O_3_	2%	162	161, 146, 133	[60,61]
3	Sinapoylquinic acid isome	6.1	C_18_ H_22_ O_10_	2.5%	397	397, 232, 166	[62]
4	Quercetin 5,3’,4’-trimethyl ether 3-galactosyl-(1->2)-rhamnoside-7-rhamnoside	6.4	C_36_H_46_O_20_	4.5%	797	797, 284	[63]
5	3,7-Dimethylquercetin	9.4	C_17_H_14_O_7_	6%	329	328, 302, 287	[64,65]
6	*p*-Coumaroyl tartaric acid	15.2	C_13_H_12_O_8_	7%	295	294, 263, 251	[56]
7	Hesperidin	15.3	C_28_H_34_O_15_	5%	610	610, 600, 163	[66]
8	*p*-Coumaroyl tartaric acid	16.7	C_13_H_12_O_8_	1%	295	294, 263, 251	[56]
9	Naringenin	18.9	C_15_H_14_O_5_	4%	271	271, 243, 230	[67]
10	1,2-Benzopyrone(coumarin)	29.8	C_9_H_6_O_2_	1%	145	145, 116	[68]

**Table 3:** Lipinski’s rule of five for ADME analysis of Xanthotoxin.

No.	Name	M.wt	Lipophilicity	Hydrogen Bond Donors	Hydrogen Bond Acceptors	No. of Rule Violations	Drug-Likeness
		Less than 500 g/mol	Less than 5	Less than 5	Less than 10	Less than 2 Violations	Lipinski’s Rule Follows Rule
1	Xanthotoxin	216.19	1.18	0	4	0	Yes

**Table 4:** Comparison between Group I (control) and Group 2 (experimental) according to keratin thickness in (fungiform) and (filiform).

	Group 1, µm	Group 2, µm	*t*-Test	*p*-Value
Keratin Thickness (Fungiform)				
Mean ± SD	51.40 ± 0.22	9.40 ± 0.22	363.731	<0.001 **
Range	51.1–51.7	9.1–9.7		
Keratin Thickness (Filliform)				
Mean ± SD	51.60 ± 0.22	9.60 ± 0.22	341.696	<0.001 **
Range	51.3–51.9	9.3–9.9		

Data are expressed as mean ± SD; t independent sample *t*-test for mean ± SD; ** *p*-value < 0.001 is highly significant.

## Data Availability

Data are available in the manuscript.
